# Perinatal colonization with extended-spectrum beta-lactamase-producing and carbapenem-resistant Gram-negative bacteria: a hospital-based cohort study

**DOI:** 10.1186/s13756-024-01366-9

**Published:** 2024-01-29

**Authors:** Ashley Styczynski, Mohammed Badrul Amin, Kazi Injamamul Hoque, Shahana Parveen, Abu Faisal Md Pervez, Dilruba Zeba, Akhi Akhter, Helen Pitchik, Mohammad Aminul Islam, Muhammed Iqbal Hossain, Sumita Rani Saha, Emily S. Gurley, Stephen Luby

**Affiliations:** 1 Division of Infectious Diseases and Geographic Medicine, Stanford University, Palo Alto, CA USA; 2 Laboratory of Food Safety and One Health, Laboratory Sciences and Services Division, icddr,b, Dhaka, Bangladesh; 3 Programme on Emerging Infections, icddr,b, Dhaka, Bangladesh; 4 Department of Pediatrics, Bangabandhu Sheikh Mujib Medical College, Faridpur, Bangladesh; 5 Department of Obstetrics and Gynaecology, Bangabandhu Sheikh Mujib Medical College, Faridpur, Bangladesh; 6 Division of Epidemiology, School of Public Health, University of California Berkeley, Berkeley, CA USA; 7 Paul G. Allen School for Global Health, Washington State University, Pullman, WA USA; 8 Department of Epidemiology, Johns Hopkins University, Baltimore, MD USA

**Keywords:** Antimicrobial resistance, Perinatal, Colonization, Hospital, Delivery

## Abstract

**Background:**

Antimicrobial resistance (AMR) is a growing global health threat that contributes to substantial neonatal mortality. Bangladesh has reported some of the highest rates of AMR among bacteria causing neonatal sepsis. As AMR colonization among newborns can predispose to infection with these bacteria, we aimed to characterize the frequency of and risk factors for colonization of mothers and newborns during hospitalization for delivery.

**Methods:**

We enrolled pregnant women presenting for delivery to a tertiary care hospital in Faridpur, Bangladesh. We collected vaginal and rectal swabs from mothers pre- and post-delivery, rectal swabs from newborns, and swabs from the hospital environment. Swabs were plated on agars selective for extended-spectrum-beta-lactamase producing bacteria (ESBL-PB) and carbapenem-resistant bacteria (CRB). We performed logistic regression to determine factors associated with ESBL-PB/CRB colonization.

**Results:**

We enrolled 177 women and their newborns during February-October 2020. Prior to delivery, 77% of mothers were colonized with ESBL-PB and 15% with CRB. 79% of women underwent cesarean deliveries (C-section). 98% of women received antibiotics. Following delivery, 98% of mothers and 89% of newborns were colonized with ESBL-PB and 89% of mothers and 72% of newborns with CRB. Of 290 environmental samples, 77% were positive for ESBL-PB and 69% for CRB. Maternal pre-delivery colonization was associated with hospitalization during pregnancy (RR for ESBL-PB 1.24, 95% CI 1.10–1.40; CRB 2.46, 95% CI 1.39–4.37). Maternal post-delivery and newborn colonization were associated with C-section (RR for maternal CRB 1.31, 95% CI 1.08–1.59; newborn ESBL-PB 1.34, 95% CI 1.09–1.64; newborn CRB 1.73, 95% CI 1.20–2.47).

**Conclusions:**

In this study, we observed high rates of colonization with ESBL-PB/CRB among mothers and newborns, with pre-delivery colonization linked to prior healthcare exposure. Our results demonstrate this trend may be driven by intense use of antibiotics, frequent C-sections, and a contaminated hospital environment. These findings highlight that greater attention should be given to the use of perinatal antibiotics, improved surgical stewardship for C-sections, and infection prevention practices in healthcare settings to reduce the high prevalence of colonization with AMR organisms.

**Supplementary Information:**

The online version contains supplementary material available at 10.1186/s13756-024-01366-9.

## Introduction

Antimicrobial resistance (AMR) is a growing global health threat that disproportionately affects low- and middle-income countries (LMIC) and represents one of the leading causes of mortality worldwide [1, 2]. Neonates are a key risk group for infections with AMR organisms given their immature microbiome and underdeveloped host defenses [3, 4]. Neonates are often exposed to a wide range of bacteria at the time of birth, which can result in colonization. Colonization occurs when bacteria persist in or on body surfaces without causing illness. Although colonization does not directly cause disease, colonization with resistant bacteria can predispose individuals to developing drug-resistant infections, particularly among newborns with prematurity and low birthweight [5–7]. Neonatal sepsis caused by AMR organisms results in higher rates of mortality compared with non-AMR infections [8]. An estimated 200,000 neonatal deaths annually have been attributed to infections with AMR organisms [1, 9].

In many LMICs, the majority of cases of neonatal sepsis are caused by Gram-negative bacteria, many of which are multidrug resistant [8, 10–13]. A report from 2020 demonstrated that 81% of Gram-negative bacteria causing sepsis in newborns across three neonatal care units in Bangladesh were resistant to carbapenems, one of the last line antibiotic options [12]. Similar concerning trends of increasing AMR in neonatal infections have been observed elsewhere in South and Southeast Asia, including among homebirths, demonstrating an increasing community reservoir for AMR [14–16].

Healthcare facilities, in particular, have been implicated as an important source of AMR amplification because of the associated intense antibiotic use and admixing of ill and susceptible patients [17, 18]. This is further exacerbated in low-resource hospitals because of overcrowding, understaffing, inadequate hygiene and sanitation, and a lack of access to diagnostics. In these settings, antibiotics are often used liberally in healthcare facilities as a substitute for improved hygiene and sanitation, and the lack of diagnostics precludes antibiotic stewardship practices [19, 20]. Understanding the environments that are promoting evolution and transmission of such organisms to newborns is an essential step towards preventing exposure, thereby avoiding downstream consequences such as resistant infections that may be difficult or impossible to treat.

The objective of this study was to estimate the burden of and risk factors for colonization with extended-spectrum beta-lactamase producing bacteria (ESBL-PB) and carbapenem-resistant bacteria (CRB) among mothers and newborns in the context of facility-based deliveries. As one step in the infection pathway, colonization provides a useful parameter for monitoring transmission patterns that could predispose to infection [21–23].

## Methods

### Participant enrollment

**Fig. 1:**
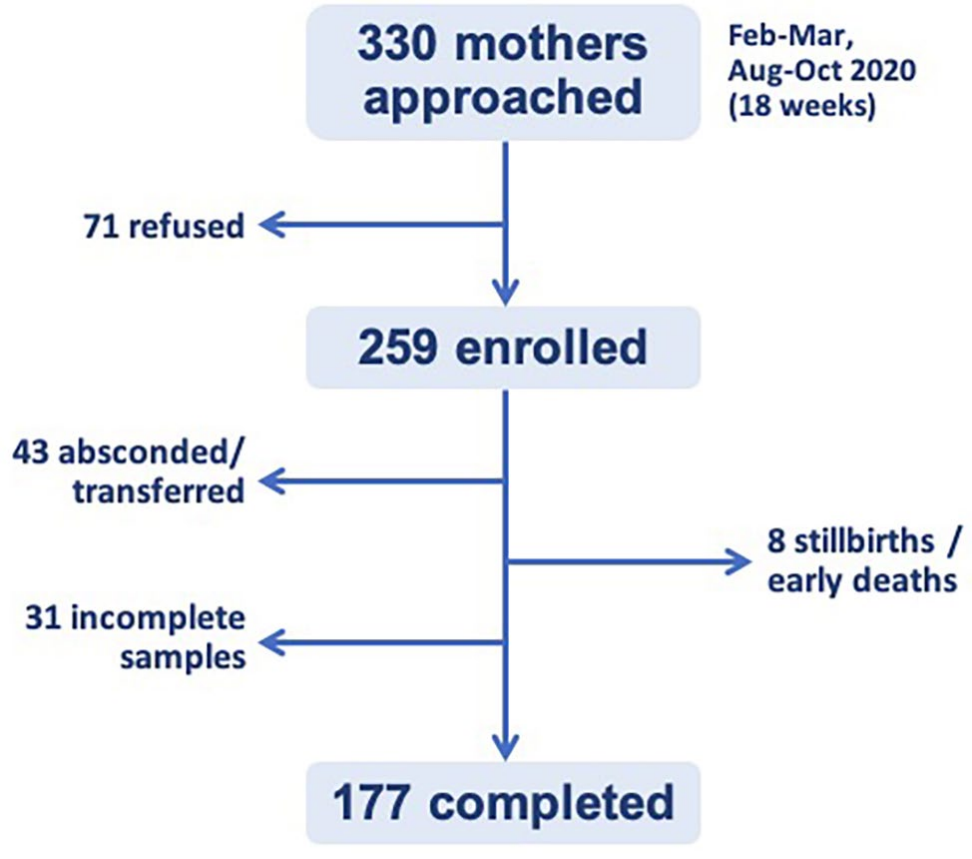
**Study enrollment of pregnant mothers presenting for delivery at a tertiary care facility, Faridpur, Bangladesh, 2020**. Flowchart of number of pregnant women approached for enrollment, number enrolled, and number who completed all surveys and specimen collections, as well as the reasons for attrition

We enrolled pregnant women presenting for delivery at a tertiary care public medical college hospital in Faridpur, Bangladesh, during February – March and August – October, 2020. A four-month interruption in enrollment (April-July) occurred as a result of data collection restrictions during the COVID-19 pandemic. A trained member of the nursing staff collected a set of vaginal and rectal swabs. The project research physician conducted interviews with the participants to obtain demographic and community exposure information about exposures that were hypothesized could be related to AMR colonization, including socioeconomic status, sanitation, animal contact, antibiotic use, and healthcare contact (S1 Fig). Over the course of the hospitalization, the research physician gathered information from the medical charts and care providers regarding treatments and interventions. Prior to hospital discharge, and at least 24 h after delivery, the nursing staff collected a second set of vaginal and rectal swabs from the mothers and a rectal swab from their newborns. A total of 177 mother/baby pairs completed data collection (Fig. 1).

### Environmental sampling

During the same time interval as participant enrollment, the study team collected samples of the hospital environment from the perinatal ward, labor room, and the operating room in which cesarean deliveries (C-sections) were performed. The selection of sampled items was determined following a three-day observation period during which frequently touched surfaces and shared equipment were identified and counts of hand contacts were noted for each type of surface or equipment. We purposefully sampled these sites throughout the study period. The sampling sites included hands of healthcare workers and patient attendants, shared medical equipment, beds, faucets, doors, toilet facilities, oxygen delivery devices, floors, and other surfaces. For each day of participant enrollment, we obtained three environmental swabs. Swabs were first moistened with sterile water before sampling surfaces.

### Bacterial culturing of swab samples

Participant (rectal and vaginal) and environmental swabs were placed in Amies media and stored at 2–8 °C until they could be transported to the lab. All swabs were processed within 24 h of collection. Participant swabs were directly inoculated onto CHROMagar ESBL and CHROMagar mSuperCARBA (CHROMagar, Paris, France). Environmental swab samples were enriched with trypticase soy broth (TSB) with overnight incubation at 37 °C before the samples were inoculated onto the same media as the participant swabs. From the chromogenic agars, bacterial colony growth color and characteristics were recorded after overnight incubation at 37 °C.

### Statistical analysis

We performed descriptive statistics to summarize epidemiologic characteristics of participants. We compared the proportion of colonized individuals and environmental samples before the start of the COVID-19 pandemic (Feb-Mar) and during the pandemic (Aug-Oct). To examine the relationship between community-based exposures on pre-delivery AMR colonization patterns and hospital-based exposures on post-delivery AMR colonization patterns, we performed logistic regression. We calculated unadjusted risk ratios for all community- and hospital-based exposures. The sample size for the risk factor analysis was determined using an estimated 80% colonization with at least one AMR organism and a 20% expected difference in outcomes between exposed and unexposed individuals, considering a design effect of 1.2. We used McNemar’s test to determine differences in colonization prevalence across various time points for paired samples and a test of proportions to determine differences in colonization for unpaired samples.

Community or hospital exposures with *p*-values < 0.2 that had at least 10% prevalence and were not collinear were included in multivariable analysis. In the newborn analyses, we did not control for maternal post-delivery colonization to avoid masking potentially significant newborn exposures. No additional model refining was performed as the objective of the analysis was not to optimize a final predictive model, which would likely not be robust given the limited dataset, but to generate hypotheses regarding factors most likely to drive AMR colonization. Participants with missing data were excluded from the respective analyses, which only included age (eight participants) and income (one participant). All analyses were conducted using the statistical program Stata (Version 17.0, StataCorp, College Station, TX).

## Results

### Demographics of pregnant women enrolled in the study

Of the 177 women enrolled in the study, the median age was 25 years (range 17–40) (Table 1). The majority of participants (73%) had at least a secondary school education. The median monthly household income was 201 USD (amounting to $1.34/person/day). Almost all households had improved drinking water sources, with 98% of households reporting tube wells as the main water source. Pit latrines were the most common toilet type (79%). Approximately one-third (33%) of the women had no prior pregnancies. While most women had received some prenatal care, only 14% reported four or more visits. Anemia was the most frequent pregnancy complication, reported by nearly half of participants. Two-thirds of deliveries occurred at or post-term.

**Table 1:** Community-based characteristics of pregnant mothers presenting for delivery, Faridpur, Bangladesh, 2020 (*N* = 177)

	n	% or range
Number of women who completed the study	177	
Number of babies	179	
Median age of women*	25	17–40
Highest education completed		
No formal education	12	7%
Primary school	35	20%
Secondary school	112	63%
Bachelor’s degree	10	6%
Master’s degree	8	5%
Occupation**		
Homemaker	174	98%
Poultry/livestock rearer	14	8%
Student	6	3%
Domestic animal contact during pregnancy	177	100%
Median monthly income (USD)***	201	60–885
Median household size	5	2–8^+^
Main water source – tube well	173	98%
Toilet type		
Shared pit latrine	82	46%
Private pit latrine	57	32%
Flush/pour flush toilet	38	22%
Nulliparous at enrollment	58	33%
Antenatal care visits		
None	35	20%
1–3 visits	117	66%
4 or more visits	25	14%
Pregnancy complications (self-reported)		
Anemia	87	49%
Urinary infection	32	18%
Bleeding	17	10%
High blood pressure	14	8%
Antibiotic use within 30 days prior to admission	23	13%
Hospitalization during pregnancy prior to delivery	21	12%
Timing of delivery		
Preterm (< 37 weeks)	54	31%
Full term (37–42 weeks)	110	62%
Post term (> 42 weeks)	13	7%

*N = 169. Those without a reported age were excluded from the median age calculation

**Occupation categories are not mutually exclusive as women were asked to report all occupations

***N = 176. The individual without income data available was excluded from the median income calculation

The majority of women (79%) delivered via C-section (Table 2). Duration of hospitalization was longer for women undergoing C-section compared with vaginal delivery (4.0 versus 1.6 days, *p* < 0.001). Nearly all women received perinatal prophylactic antibiotics (98%), of whom, 91% received them before or during delivery. The prescribed duration of antibiotics was longer for women who underwent C-section compared with vaginal delivery (10 versus 7.6 days, *p* < 0.001). The most common antibiotics administered were metronidazole (89%), flucloxacillin (69%), and third-generation cephalosporins (ceftriaxone or cefixime) (67%) (S1 Table). The most frequent antibiotic regimen was a combination of a third-generation cephalosporin, metronidazole, and flucloxacillin (48%). No carbapenem use was reported. More than half of the participants experienced a pregnancy complication, including fetal distress/cord prolapse (40%), obstructed/prolonged labor (27%), or prolonged rupture of membranes (> 24 h) (23%).

**Table 2:** Hospital-based characteristics and management of pregnant mothers presenting for delivery, Faridpur, Bangladesh, 2020 (*N* = 177)

	n	% or range
Labor management		
Membrane sweeping/stripping	50	28%
6 or more vaginal exams	45	25%
Artificial rupture of membranes	19	11%
Mechanical cervical ripening	14	8%
Mode of delivery		
Vaginal delivery	37	21%
Cesarean delivery	140	79%
Mother received antibiotics	174	98%
Duration of antibiotics, median (days)*	10	3–15
Timing of antibiotic initiation*		
Before/during delivery	158	91%
After delivery	16	9%
Indication for antibiotics*		
Prevention	174	100%
Treatment	1	1%
Delivery complications		
Fetal distress/cord prolapse	70	40%
Obstructed/prolonged labor	48	27%
Prolonged rupture of membranes	41	23%
Retained placenta	7	6%
Duration of hospitalization, median (days)	3	1–9
Admission to ICU	9	5%

*N = 174, the number who received antibiotics; ICU: Intensive care unit

Among the newborns, nearly all received airway clearing, wiping and wrapping, temperature and weight measuring, and feeding initiation within the first hour of birth (Table 3). Approximately one-quarter underwent resuscitation with two newborns requiring artificial ventilation. 5% of newborns received antibiotics, including three (2%) newborns with clinically-diagnosed neonatal sepsis. No blood cultures were collected at the time of diagnosis or during the course of treatment. No neonatal deaths occurred among enrolled newborns during the course of the study.

**Table 3:** Hospital-based characteristics and management of newborns delivered at a tertiary care facility, Faridpur, Bangladesh, 2020 (*N* = 177*)

	n	% (range)
Female	82	46%
Birthweight, median (g)	2900	1500–4200
Immediate management of newborn (within 1 h of birth)		
Cleared airway	176	99%
Wiped and wrapped	176	99%
Temperature and weight measured	173	98%
Initiated feeding	171	97%
Administered vitamin K	98	55%
Resuscitated	40	23%
Mechanical ventilation	2	1%
Newborn received antibiotics	9	5%
Duration of antibiotics, median (days)**	3	1–5
Indication for antibiotics**		
Prevention	8	89%
Treatment	1	11%
Newborn sepsis	3	2%
Duration of hospitalization, median (days)	3	1–7
Admission to neonatal ICU	9	5%

*For the two sets of twins, one twin was randomly excluded from each set for analysis purposes

**N = 9, the number of newborns who received antibiotics

### Colonization patterns of mothers and newborns with ESBL-PB and CRB

**Fig. 2:**
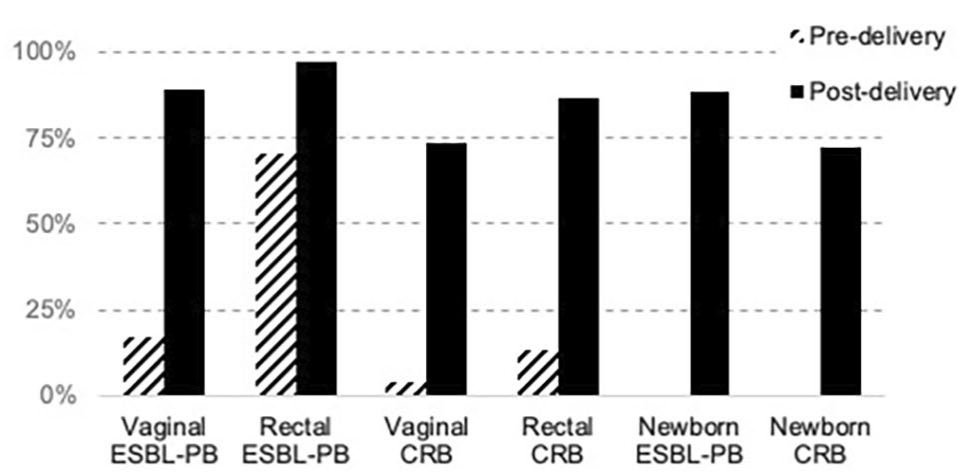
**Vaginal and rectal colonization of mothers and rectal colonization of newborns with ESBL-producing and carbapenem resistant bacteria at a tertiary care facility, Faridpur, Bangladesh, 2020** (***N* = 177).** Prevalence of colonization patterns among mothers and newborns. Colonization was compared pre- and post-delivery to determine differences between community-based colonization and colonization following healthcare exposure. ESBL-PB = organisms recovered from agar selective for extended-spectrum beta-lactamase-producing bacteria; CRB = organisms recovered from agar selective for carbapenem resistant bacteria. ***McNemar’s test *p* < 0.001

On admission, 17% of women (*n* = 30) had vaginal colonization and 71% (*n* = 125) had rectal colonization with organisms recovered from CHROMagar ESBL plates (hereafter referred to as ESBL-PB), but only 15% (*n* = 27) had rectal or vaginal colonization with organisms recovered from CHROMagar mSuperCARBA plates (hereafter referred to as CRB) (Fig. 2). At the time of discharge following delivery, nearly all women had either rectal or vaginal colonization with ESBL-PB (98%, *n* = 174), 86% (*n* = 153) had rectal CRB colonization, and 74% (*n* = 130) had vaginal CRB colonization. Newborns demonstrated rectal colonization patterns similar to maternal colonization patterns on discharge: 89% (*n* = 157) were colonized with ESBL-PB, and 72% (*n* = 128) were colonized with CRB.

### Prevalence of ESBL-PB and CRB in environmental samples

**Fig. 3:**
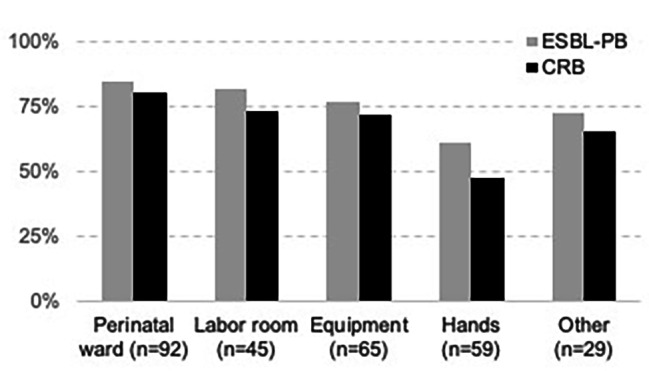
**Environmental detection of ESBL-producing and carbapenem resistant bacteria in the obstetric facilities of a tertiary care hospital, Faridpur, Bangladesh, 2020** (***N*** **= 290).** Frequency of contamination of various hospital environmental surfaces during the study period. Hand samples were taken from healthcare workers as well as patient attendants. A full list of sampled surfaces is in S3 Table. ESBL-PB = organisms recovered from agar selective for extended-spectrum beta-lactamase-producing bacteria; CRB = organisms recovered from agar selective for carbapenem resistant bacteria

A total of 290 environmental swab samples were collected from the perinatal ward, labor room, and operating room (S2 Table). Overall, 77% (*n* = 222) of samples were positive for ESBL-PB and 69% (*n* = 201) were positive for CRB; patterns were similar across types of samples analyzed (Fig. 3). However, many surfaces had multiple colony morphologies on the selective plates, demonstrating broad organism diversity (S2 Fig).

### Prevalence of ESBL-PB/CRB colonization in relation to COVID-19

Pre-delivery rectal ESBL-PB colonization was significantly higher after the start of the COVID-19 pandemic (81%) compared with before COVID (63%, *p* = 0.01) (S3 Table). Similarly, pre-delivery rectal CRB colonization increased during COVID (8% vs. 20%, *p* = 0.02). Pre-delivery vaginal ESBL-PB and CRB colonization also increased, but the differences were not significant (ESBL-PB: 15% vs. 20%, *p* = 0.43; CRB: 2% vs. 6%, *p* = 0.13).

In contrast, post-delivery rectal colonization was similar before and after the start of COVID (ESBL-PB: 96% vs. 99%, *p* = 0.28; CRB 83% vs. 91%, *p* = 0.13). Post-delivery vaginal colonization was also stable (ESBL-PB: 92% vs. 86%, *p* = 0.18; CRB: 72% vs. 75%, *p* = 0.62). Newborn colonization increased, but the differences were only marginally significant (ESBL-PB: 85% vs. 94%, *p* = 0.08; CRB: 67% vs. 79%, *p* = 0.07).

Overall environmental contamination with ESBL-PB and CRB was markedly higher during COVID compared with before the pandemic (ESBL-PB: 54% vs. 94%, *p* < 0.001; CRB: 45% vs. 89%, *p* < 0.001) (S3 Figure).

### Community and hospital exposures associated with ESBL-PB/CRB colonization in mothers and newborns

Colonization outcome variables were grouped by resistance phenotype as the selective pressures contributing to colonization with a given phenotype are likely to be similar regardless of body site. Although vaginal colonization with AMR organisms was generally lower than rectal colonization, this is likely a result of different microbiome constitutions, with a predominance of Gram-negative organisms colonizing the rectum compared with the vaginal canal.

In the multivariate analysis of community-based exposures, variables associated with an increased risk for pre-delivery ESBL-PB colonization included prior hospitalization during pregnancy (RR 1.24, 95% CI 1.10–1.40) and preterm delivery (1.18, 95% CI 1.02–1.37) (Table 4). Variables associated with pre-delivery CRB colonization were seven or more people living in the household (RR 2.39, 95% CI 1.11–5.11), goats outside the home (RR 0.44, 95% CI 0.24–0.80), prior hospitalization during pregnancy (RR 2.46, 95% CI 1.39–4.37), and antibiotic use within the prior 30 days (RR 2.51, 95% CI 1.45–4.35). While it did not reach statistical significance at a *p* < 0.05 cutoff, raising ducks outside the home had the highest measures of association with pre-delivery CRB colonization (RR 6.36, 95% CI 0.91–44.3).

**Table 4:** Association of community-based exposures with maternal pre-delivery ESBL-producing and carbapenem resistant bacteria vaginal and rectal colonization, Faridpur, Bangladesh, 2020

Pre-delivery ESBL-PB				Pre-delivery CRB			
	RR	95% CI	*p*-value		RR	95% CI	*p*-value
Hospitalization during pregnancy	**1.24**	**1.10–1.40**	**< 0.001**	Number of people living in the household			
Preterm delivery	**1.18**	**1.02–1.37**	**0.02**	2–4	Ref		
				5–6	1.00	0.46–2.15	0.99
				7 or more	**2.39**	**1.11–5.11**	**0.03**
				Ducks outside the house	6.36	0.91–44.3	0.06
				Goats outside the house	**0.44**	**0.24–0.80**	**0.01**
				Hospitalization during pregnancy	**2.46**	**1.39–4.37**	**0.002**
				Antibiotic use in the 30 days prior to delivery	**2.51**	**1.45–4.35**	**0.001**

Multivariable regression analyses include all non-collinear variables with *p* < 0.2 and at least 10% prevalence. Results in bold have *p* < 0.05 level. RR = relative risk; ESBL-PB = organisms recovered from agar selective for extended-spectrum beta-lactamase-producing bacteria; CRB = organisms recovered from agar selective for carbapenem resistant bacteria

Although the prevalence was too low to allow inclusion in the multivariate analysis, other significant variables associated with ESBL-PB colonization in the bivariate analysis included tending livestock or poultry (RR 1.23, 95% CI 1.04–1.46), having a private pit latrine compared with a shared pit latrine (RR 0.79, 95% CI 0.64–0.97), and straining drinking water through cloth (RR 1.23, 95% CI 1.04–1.46) (S5 Table). Factors associated with CRB colonization were tending livestock or poultry (RR 4.08, 95% CI 2.09–7.94), storing water in a container with a wide opening compared with a narrow opening (RR 0.33, 95% CI 0.12–0.95), and being underweight (RR 4.64, 95% CI 1.92–11.2).

Among hospital exposures, no variables were associated with post-delivery maternal ESBL-PB colonization at a *p* < 0.2 cutoff, so no multivariable model was created. Factors associated with post-delivery maternal CRB colonization included undergoing C-section (RR 1.31, 95% CI 1.08–1.59) and experiencing complications at the time of delivery (RR 1.13, 95% CI 1.03–1.24) (Table 5). Because duration of hospitalization was collinear with type of delivery, it was not included in the multivariable analysis. Similarly, antibiotics administered to mothers before delivery was also collinear with type of delivery and was thus excluded from multivariable analysis. We did not control for colonization pre-delivery as most mothers with CRB colonization post-delivery did not have colonization pre-delivery.

**Table 5:** Association of hospital-based exposures with maternal and newborn colonization with ESBL-producing and carbapenem resistant bacteria

Post-delivery CRB				Newborn ESBL-PB			
	RR	95% CI	*p*-value		RR	95% CI	*p*-value
Delivery mode				Delivery mode			
Vaginal delivery	Ref			Vaginal delivery	Ref		
Cesarean delivery	**1.31**	**1.08–1.59**	**0.01**	Cesarean delivery	**1.34**	**1.09–1.64**	**0.01**
Maternal complications at the time of delivery	**1.13**	**1.03–1.24**	**0.01**	Maternal pre-delivery CRB colonization	**1.15**	**1.09–1.21**	**< 0.001**
Mother received third-generation cephalosporin antibiotics	0.96	0.93-1.00	0.05				

Multivariable regression analyses include all non-collinear variables with *p* < 0.2 and at least 10% prevalence. Maternal colonization post-delivery was not included in newborn results to avoid controlling for maternal factors that could mask associations. Results in bold have *p* < 0.05 level. RR = relative risk; ESBL-PB = organisms recovered from agar selective for extended-spectrum beta-lactamase-producing bacteria; CRB = organisms recovered from agar selective for carbapenem resistant bacteria

Newborn ESBL-PB colonization was also associated with C-section (RR 1.34, 95% CI 1.09–1.64) and maternal pre-delivery CRB colonization (RR 1.15, 95% CI 1.09–1.21). The only factor associated with newborn CRB colonization at a *p* < 0.2 cutoff was delivery by C-section (RR 1.73, 95% CI 1.20–2.47) (S7 Table). Although not included in the multivariate analysis, newborn CRB colonization was significantly associated with maternal post-delivery CRB colonization (RR 2.45, 95% CI 1.25–4.77). No association was found with maternal post-delivery ESBL-PB colonization.

## Discussion

This study revealed a high prevalence of perinatal colonization with ESBL-PB and CRB among mothers and newborns undergoing facility-based deliveries at a tertiary care hospital in Bangladesh. Colonization prevalence was substantially higher in mothers at the time of discharge compared with admission, particularly for CRB. The less notable changes in ESBL-PB colonization may be attributable to the frequent ESBL-PB colonization on admission. Additionally, there were concurrently high rates of C-sections and frequent prescribing of prolonged courses of prophylactic antibiotics.

Newborn colonization prevalence more closely resembled maternal colonization at discharge compared with admission and occurred within the context of widespread environmental contamination. A similarly high prevalence of ESBL-PB colonization in newborns has been reported from studies in India, Cambodia, Madagascar, and Tanzania, though CRB colonization was infrequent [24–27]. Another study from Bangladesh revealed that 82% of healthy infants were colonized with *Escherichia coli* resistant to third-generation cephalosporins [28]. Much lower neonatal AMR colonization rates have been reported in high-income countries such as Israel and Sweden where ESBL-PB colonization ranged from 5 to 14% [29, 30]. This may reflect differences in local epidemiology as well as facility-based practices. This study has revealed one of the highest reported prevalences of CRB colonization among newborns, which is particularly concerning given the lack of effective treatment options for infections with these organisms. It is likely these organisms are leading to infections given studies showing high levels of CRBs causing neonatal sepsis in Bangladesh [12]. Furthermore, the remarkably high prevalence of CRB colonization occurred despite no reported use of carbapenem antibiotics, demonstrating that other beta-lactam antibiotics may be promoting CRB colonization [31, 32].

This particular hospital is a tertiary care facility where many high-risk pregnancies are referred, which may partially explain the high rates of C-sections. However, this also reflects national trends in Bangladesh. In 2018, one-third of deliveries occurred by C-section, including 67% of facility-based deliveries [33]. This is well above the 10–15% recommendation by WHO for rates of C-section [34]. The WHO threshold is based on a review of data demonstrating no improvement in perinatal mortality for higher rates of C-section [35]. This trend of increasing C-sections is growing fastest in LMICs [36]. Rather than reflecting improved global access to a life-saving procedure, the disparities in rates of C-sections – from 5% in sub-Saharan Africa to 43% in Latin America and the Caribbean – reveal ongoing incongruencies and inappropriate surgical stewardship. By 2030, nearly a third of all deliveries globally are expected to occur by C-section, with the vast majority (nearly 90%) occurring in LMICs [36].

Along with frequent C-sections, perinatal antibiotic use was high among participants. Despite WHO guidance recommending only a single dose of pre-operative prophylactic antibiotics for C-sections, most participants who underwent C-section were advised to complete prolonged courses of antibiotics [37]. Moreover, the WHO guidance emphasizes the use of narrow-spectrum antibiotics for prophylaxis. Reports from India demonstrate prolonged courses of prophylactic antibiotics being given to 80% of women undergoing C-sections, including frequent use of three-drug regimens [38]. A study from China revealed that 100% of women who underwent C-sections received a standard seven-day antibiotic regimen [39]. Similar practices have been reported from other LMICs, indicating the use of prolonged prophylactic antibiotics for C-sections may be widespread, despite evidence showing no benefit from multiple doses of antibiotics [40–43]. This contrasts with practices in high-resource contexts, such as the U.S., where multiple doses of antibiotics are rare [44, 45].

Although the analysis conducted here was exploratory, prior hospitalization was consistently associated with increased risk for maternal ESBL-PB/CRB colonization, implicating the role of healthcare settings in the propagation of AMR. Additionally, there was an association between preterm delivery and maternal vaginal ESBL-PB colonization. This suggests that the vaginal microbiome may be having an effect on preterm delivery, which has been previously demonstrated [46, 47]. It remains unclear what the mechanism is for how colonization with resistant organisms modulates preterm delivery. However, it has been proposed that antibiotic resistant bacteria may be associated with more inflammation and perhaps reflective of dysbiosis [48, 49]. Further studies are needed to characterize this potential dynamic.

Animal contact appears to be connected with colonization. Duck rearing was associated with increased pre-delivery maternal CRB colonization risk, though the relatively low number of participants with this exposure likely resulted in it not being statistically significant. Similarly, tending livestock or poultry was associated with pre-delivery ESBL-PB and CRB colonization in bivariate analysis but was not included in the multivariate analysis because so few participants had this exposure. The only other significant association with animals was an apparent protective effect of goats outside the house. These findings may warrant further investigation into the One Health processes that could be contributing to AMR propagation and dissemination.

C-section was associated with post-delivery maternal CRB colonization as well as newborn ESBL-PB/CRB colonization. Other factors commonly associated with AMR such as length of hospital stay and antibiotic administration could not be included in the multivariate analyses because these were collinear with mode of delivery. This is consistent with another study in a low-resource context examining associations with neonatal AMR colonization that found aspects of the healthcare setting to be the most highly associated factors, including antibiotic use, longer hospital stays, prematurity, and lower staffing ratios [50]. Additionally, C-sections have been shown to lead to a disrupted colonizing microbiota among neonates [51–53], which may be exacerbated by antibiotic use [54]. Even when the antibiotics are only administered to the mother, studies have demonstrated negative health outcomes in the newborn, including necrotizing enterocolitis [55], neonatal sepsis [56], and growth stunting [54]. Further, the magnitude of disruption on the neonatal microbiome attributable to intrapartum antibiotics administered to mothers has been found to be similar to postnatal antibiotics administered to newborns directly [57]. Thus, ESBL-PB and/or CRB colonization may be a marker of dysbiosis, induced by upstream factors such as antibiotic exposure that provide an opportunity for colonization with resistant bacteria [49, 58]. Accordingly, fecal microbiota transplantation has been tried as a successful strategy for restoring the microbiota in neonates born by C-section [59]. In this study, the impact of maternal antibiotic use on newborn colonization could not be assessed since nearly all women received perinatal antibiotics, with the majority of antibiotic courses started prior to delivery.

While there are likely many aspects of the healthcare setting contributing to ESBL-PB/CRB colonization, both antibiotic use and C-section rates are potentially modifiable. Apart from contributing to AMR, unnecessary C-sections may have additional negative health ramifications, such as surgical site infections, blood clots, or injury to other organs. A review of indications for C-sections in Bangladesh found that the majority were not performed out of medical necessity [60]. This is a clear area for ongoing surveillance and changes to current practices.

Overuse of antibiotics is a known driver of AMR. The prolonged antibiotic courses received by the majority of mothers for the purposes of prophylaxis pose a threat to community rates of AMR through direct and indirect effects on resistance [61]. The situation in the delivery ward is unlikely to be unique in Bangladesh given high levels of antibiotic resistance reported across a variety of settings [62, 63]. Reasons for liberal antibiotic use in Bangladesh include lack of understanding of antibiotic function, low awareness of antibiotic resistance, overemphasis on use of antibiotics for prevention, and a perception of antibiotics as a symbol of power [64]. Additionally, this tertiary care facility does not have a functioning microbiology laboratory or infectious disease consultant, limiting the ability to select appropriate antibiotics based on antibiograms or culture results or seek expert consultation.

The high level of antibiotic use for prophylaxis also points to a heightened concern for healthcare-associated infections (HAI) [65]. Hospital-acquired neonatal infections are up to 20 times more common in LMICs compared with high-income contexts [66]. Accordingly, Zaidi, et al. note in the context of LMICs, any newborn infection in a hospital-born baby should be considered a HAI, regardless of the timing of onset [66]. In LMICs such as Bangladesh, antibiotics are a readily-available tool to mitigate HAIs, certainly more accessible than infrastructure changes to improve hygiene and sanitation or even incentives for hospital workers to prioritize cleanliness. In this scenario, antibiotics are likely acting both as a substitute for and an extension of infection prevention and control efforts [20]. In fact, a prior study from Bangladesh demonstrated that antibiotics given to patients on admission resulted in less hospital-onset diarrhea resulting from contaminated food [67].

Simultaneous recovery of abundant ESBL-PB/CRB from the hospital environment further suggests that the environment is likely contributing to AMR transmission. This has been corroborated by other studies in similar contexts that have shown that surface colonization predicts infecting organisms in neonates [68, 69]. Antibiotic-resistant *Klebsiella pneumoniae* strains isolated from newborns hospitalized in a neonatal intensive care unit were found to be genetically closely related to strains isolated from other infants on the ward, supporting the notion of transmission being driven by healthcare workers and/or shared equipment [50]. Another study found that while *K. pneumoniae* transmission to newborns appears to be driven by direct contact with colonized healthcare workers, spread of *E. coli* and *Enterobacter cloacae* are mediated through indirect contamination [25]. Further supporting the linkage between exposure to the healthcare environment and ESBL-PB/CRB colonization is the increased environmental contamination during COVID-19 and concomitant increase in neonatal AMR colonization. Increased environmental contamination during COVID-19 may have resulted from decreased routine cleaning by staff who were concerned about heightened exposure to SARS-CoV-2 in the hospital setting. This supports the need for enhanced environmental cleaning and infection prevention efforts to reduce HAIs and AMR.

Some of the strengths of this study are that it employs a relatively low-cost method of AMR surveillance that could be replicated in other low-resource contexts [70]. It also uses colonization to detect AMR burden and transmission, allowing for the implementation of directed preventive measures in advance of infectious outcomes [70]. Further, the examination of both community and hospital-related factors and the measurement of colonization at two time points allows for better triangulation of the most important risk factors for AMR colonization in the same population across both settings.

This study has several limitations. The homogeneity of participants’ community and hospital exposures that we hypothesized to have strong associations with AMR colonization meant that the associations between some factors and ESBL-PB/CRB colonization could not be evaluated. However, the descriptive characteristics provide insight on common practices that may be implicated in AMR transmission, such as abundant antibiotic use. Additionally, we relied on the results of chromogenic agars for classifying ESBL-PB/CRB colonization. Prior studies using similar specimen types have reported 98–100% sensitivity for the ESBL agar and 93–100% for the mSuperCARBA agar but with lower specificities (72–100%) [71–74]. However, these studies were specifically assessing the performance of the agars for identifying ESBL-producing or carbapenemase-producing Enterobacterales. This contrasts with the objectives of this study, which were not limited only to Enterobacterales or to production of specific resistance determinants but to all Gram-negative isolates with the resistant phenotypes of interest. Moreover, we did not use the chromogenic findings as a means of definitive identification, and instead highlighted the relative changes between pre- and post-delivery as comparable and informative indicators of underlying trends.

Furthermore, we only measured colonization status, which does not in itself carry negative health consequences and may rapidly change after returning to the home environment. Yet, other studies have shown that neonatal colonization patterns can persist for up to five years, with greater persistence of more virulent bacterial strains [29, 75]. A meta-analysis examining the relationship between Gram-negative bacterial colonization and bloodstream infections in neonates did not find a statistically significant correlation, though the analysis was limited by a small number of studies to draw from and high heterogeneity between studies [6]. However, subsequent studies and other studies not included in the meta-analysis have supported the role of intestinal colonization as a predisposing factor to infection [24, 27, 68, 76].

Our findings do not prove that the environment was the source of ESBL-PB/CRB colonization among mothers and neonates as we lack bacterial characterization data. Regardless, the remarkable abundance of ESBL-PB/CRB throughout the hospital environment suggests this is likely to be a factor in at least some of the transmission pathways. Future studies including whole genome sequencing-based characterization of isolates would add further clarity to transmission dynamics and AMR diversity in this setting.

## Conclusion

The scenario of rising AMR among newborns is becoming increasingly common across LMICs and demands a close examination of the factors surrounding hospital-based deliveries, including indications for C-sections and antibiotic administration. The findings of this study highlight the need to avoid overmedicalization of deliveries. Pregnant women presenting to a hospital for delivery may be more likely to be construed as “patients in need of treatment”. Separating routine perinatal care from a healthcare facility to an adjacent birthing center may help decrease the treatment imperative. Conducting randomized controlled trials demonstrating the non-inferiority of reduced antibiotic use could further support changes in antibiotic prescribing. However, this may not be supported without concomitant improvements in infection prevention and control – with an emphasis on environmental cleaning – coupled with enhanced access to diagnostics and microbiologic laboratory capacity. Unnecessary C-sections must also be curtailed to reduce the disruption of the newborn microflora and associated morbidity. Interventions to reduce C-sections could focus on understanding and dismantling the incentives driving increasing rates of C-sections. Overall, these findings demonstrate the urgent and pressing need for better AMR surveillance and associated interventions to ensure safer birthing environments.

### Electronic supplementary material

Below is the link to the electronic supplementary material.

Supplementary Material 1

## Data Availability

All data generated or analyzed during this study are included within the article and its supplementary information files.

## Abbreviations

AMR: Antimicrobial resistance
COVID-19 (COVID): Coronavirus infectious disease 2019
CRB: Carbapenem resistant bacteria
C-section: Cesarean delivery
ESBL-PB: Extended-spectrum beta-lactamase producing bacteria
HAI: Healthcare-associated infection
ICU: Intensive care unit
LMIC: Low- and middle-income countries
