# Supplementary material for: Perinatal colonization with extended-spectrum beta-lactamase-producing and carbapenem-resistant Gram-negative bacteria: a hospital-based cohort study
Source: Antimicrob Resist Infect Control. 2024 Jan 29;13:13. doi: 10.1186/s13756-024-01366-9 (PMC10823664; doi:10.1186/s13756-024-01366-9)
Supplement: Supplementary file 1 — Supplementary Material 1 [file 13756_2024_1366_MOESM1_ESM.docx]

**Supplementary Appendix**

This appendix accompanies the following manuscript:

**Perinatal colonization with extended-spectrum beta-lactamase-producing and carbapenem-resistant Gram-negative bacteria: A hospital-based cohort study**

Ashley Styczynski, Mohammed Badrul Amin, Kazi Injamamul Hoque, Shahana Parveen, Abu Faisal Md Pervez, Dilruba Zeba, Akhi Akhter, Helen Pitchik, Mohammad Aminul Islam, Muhammed Iqbal Hossain, Sumita Rani Saha, Emily S. Gurley, Stephen Luby

Table of Contents

[Supplemental Tables 3](#_Toc144125525)

[S1 Table. Maternal antibiotics and prophylactic antibiotic regimens (N=174) 3](#_Toc144125526)

[S2 Table. Surfaces sampled for environmental swab collection 4](#_Toc144125527)

[S3 Table. Prevalence of ESBL-BP/CRB colonization before and during COVID-19 5](#_Toc144125528)

[S4 Table. Bivariate associations of community-based exposures with maternal pre-delivery ESBL-PB/CRB vaginal and rectal colonization, Faridpur, Bangladesh, 2020 6](#_Toc144125529)

[S5 Table. Bivariate associations of hospital-based exposures with maternal post-delivery ESBL-PB/CRB vaginal and rectal colonization, Faridpur, Bangladesh, 2020 12](#_Toc144125530)

[S6 Table. Bivariate associations of hospital-based exposures with newborn ESBL-PB/CRB rectal colonization, Faridpur, Bangladesh, 2020 15](#_Toc144125531)

[Supplemental Figures 18](#_Toc144125532)

[S1 Fig. Causal pathway 18](#_Toc144125533)

[S2 Fig. Bacterial growth from different environmental surfaces 19](#_Toc144125534)

[S3 Fig. Environmental contamination with ESBL-PB/CRB before and during COVID-19 19](#_Toc144125535)

### Supplemental Tables

#### S1 Table. Maternal antibiotics and prophylactic antibiotic regimens (N=174)

| **Antibiotic** | **N (%)** |
| --- | --- |
| Flucloxacillin | 120 (69%) |
| 1^st^ gen cephalosporin | 25 (14%) |
| 2^nd^ gen cephalosporin | 33 (19%) |
| 3^rd^ gen cephalosporin | 116 (67%) |
| Cephalosporin/beta-lactamase inhibitor | 2 (1%) |
| Ciprofloxacin | 1 (1%) |
| Aminoglycoside | 2 (1%) |
| Metronidazole | 154 (89%) |
| **Antibiotic Regimens** |  |
| 3^rd^ gen cephalosporin + metronidazole + flucloxacillin | 84 (48%) |
| 2^nd^ gen cephalosporin + metronidazole + flucloxacillin | 33 (19%) |
| 1^st^ gen cephalosporin + metronidazole | 25 (14%) |

Frequency of antibiotics and antibiotic regimens given to mothers during hospitalization for delivery; all 174 mothers who received antibiotics were given antibiotics for infection prophylaxis. 1^st^ generation cephalosporin includes cefradine, 2^nd^ generation cephalosporin includes cefuroxime, and 3^rd^ generation cephalosporin includes ceftriaxone and cefixime.

#### S2 Table. Surfaces sampled for environmental swab collection

| **Category** | **% Positive for ESBL-PB** | **% Positive for CRB** | **Notes** |
| --- | --- | --- | --- |
| Ward (n=36) | 86% | 81% | Nurses’ desk, doctors’ table, doors, sink faucet, patient bench, patient chair |
| Labor room (n=31) | 77% | 65% | Labor bed cover, delivery apron, delivery shoes, delivery room light, sink faucet, sink basin, doors, chair |
| OR (n=9) | 56% | 44% | Operating room table, light handle, OR shoes, IV pole, oxygen mask, gurney |
| Newborn surfaces (n=37) | 85% | 78% | Newborn scale, newborn resuscitation tray, newborn oxygen mask, newborn suction device |
| HCW hands (n=48) | 54% | 42% | Doctors, nurses, midwifes, trainees/students |
| Bed (n=26) | 88% | 85% | Bed rails, mattresses, linens |
| Floor (n=29) | 86% | 86% | Perinatal ward, labor room, stairs, dressing room |
| Attendant hands (n=11) | 91% | 73% | Patient attendants’ hands |
| Toilet (n=16) | 88% | 88% | Water tap, bodna (cleaning vessel), door |
| Gurney (n=7) | 100% | 100% | Gurneys for patient transport |
| Medicine (n=12) | 75% | 75% | Medicine bottles, medicine delivery tray |
| Equipment (n=19) | 58% | 53% | Stethoscope, IV pole, scissors, equipment tray |
| Other (n=9) | 67% | 44% | Cat (lives in perinatal ward), patient mobile phone, money, patient food table, food delivery cart |

Prevalence of environmental contamination with ESBL-PB and CRB according to surface type sampled. ESBL-PB = organisms recovered from agar selective for extended-spectrum beta-lactamase-producing bacteria; CRB = organisms recovered from agar selective for carbapenem resistant bacteria.

#### S3 Table. Prevalence of ESBL-BP/CRB colonization before and during COVID-19

|  | **Before COVID** | **During COVID** | **p-value** |
| --- | --- | --- | --- |
| Pre-vaginal ESBL-PB | 15% | 19% | 0.43 |
| Pre-rectal ESBL-PB | **63%** | **81%** | **0.01** |
| Pre-vaginal CRB | 2% | 7% | 0.13 |
| Pre-rectal CRB | **8%** | **20%** | **0.02** |
| Post-vaginal ESBL-PB | 92% | 86% | 0.18 |
| Post-rectal ESBL-PB | 96% | 99% | 0.28 |
| Post-vaginal CRB | 72% | 75% | 0.62 |
| Post-rectal CRB | 83% | 91% | 0.13 |
| Newborn ESBL-PB | 85% | 94% | 0.08 |
| Newborn CRB | 67% | 79% | 0.07 |

Comparison of colonization prevalence before and during COVID-19 using McNemar’s test. Results in bold are significant at the p<0.05 level. ESBL-PB = organisms recovered from agar selective for extended-spectrum beta-lactamase-producing bacteria; CRB = organisms recovered from agar selective for carbapenem resistant bacteria.

#### S4 Table. Bivariate associations of community-based exposures with maternal pre-delivery ESBL-PB/CRB vaginal and rectal colonization, Faridpur, Bangladesh, 2020

|  | **Pre-delivery ESBL-PB** | | | | | **Pre-delivery CRB** | | | | |
| --- | --- | --- | --- | --- | --- | --- | --- | --- | --- | --- |
|  | **non-ESBL-PB**  **(N=41)** | **ESBL-PB**  **(N=136)** |  | | | **non-CRB**  **(N=150)** | **CRB**  **(N=27)** |  | | |
|  | **n (%)** | **n (%)** | **RR** | **95% CI** | **p-value** | **n (%)** | **n (%)** | **RR** | **95% CI** | **p-value** |
| **DEMOGRAPHICS** |  |  |  |  |  |  |  |  |  |  |
| Household size |  |  |  |  |  |  |  |  |  |  |
| 2-4 | 17 (42%) | 59 (43%) | Ref |  |  | 66 (44.%) | 10 (37%) | Ref |  |  |
| 5-6 | 21 (51%) | 60 (44%) | 0.95 | 0.80-1.14 | 0.60 | 70 (47%) | 11 (41%) | 1.03 | 0.46-2.30 | 0.94 |
| 7 or more | 3 (7%) | 17 (13%) | 1.09 | 0.88-1.37 | 0.42 | 14 (9%) | 6 (22%) | 2.28 | 0.94-5.53 | 0.07 |
| Highest education |  |  |  |  |  |  |  |  |  |  |
| Primary or below | 10 (24%) | 37 (27%) | Ref |  |  | 41 (27%) | 6 (22%) | Ref |  |  |
| Secondary | 21 (51%) | 68 (50%) | 0.97 | 0.80-1.17 | 0.76 | 75 (50%) | 14 (52%) | 1.23 | 0.51-3.00 | 0.51 |
| College or above | 10 (24%) | 31 (23%) | 0.96 | 0.76-1.21 | 0.73 | 34 (23%) | 7 (26%) | 1.34 | 0.49-3.67 | 0.57 |
| Income |  |  |  |  |  |  |  |  |  |  |
| <12000 BDT | 14 (44%) | 39 (35%) | Ref |  |  | 43 (36%) | 10 (44%) | Ref |  |  |
| 12000-25000 BDT | 6 (19%) | 36 (32%) | 1.16 | 0.95-1.43 | 0.14 | 36 (30%) | 6 (26%) | 0.76 | 0.30-1.92 | 0.56 |
| >25000 BDT | 12 (38%) | 36 (32%) | 1.02 | 0.81-1.28 | 0.87 | 41 (34%) | 7 (30%) | 0.77 | 0.32-1.88 | 0.57 |
| Occupation |  |  |  |  |  |  |  |  |  |  |
| Homemaker | 41 (100%) | 133 (98%) | - |  |  | 147 (98%) | 27 (100%) | - |  |  |
| Salaried | 0 (0%) | 3 (2%) | - |  |  | 3 (2%) | 0 (0%) | - |  |  |
| Student | 1 (2%) | 5 (4%) | 1.09 | 0.75-1.57 | 0.65 | 4 (3%) | 2 (7%) | 2.28 | 0.67-7.51 | 0.18 |
| Tend livestock/poultry | 1 (2%) | 13 (10%) | **1.23** | **1.04-1.46** | **0.02** | 7 (5%) | 7 (26%) | **4.08** | **2.09-7.94** | **0.00** |
| **WATER & SANITATION** |  |  |  |  |  |  |  |  |  |  |
| Toilet type |  |  |  |  |  |  |  |  |  |  |
| Shared pit latrine | 13 (32%) | 69 (51%) | Ref |  |  | 67 (45%) | 15 (56%) | Ref |  |  |
| Private pit latrine | 19 (46%) | 38 (28%) | **0.79** | **0.64-0.97** | **0.03** | 50 (33%) | 7 (24%) | 0.67 | 0.29-1.54 | 0.35 |
| Flush/pour flush toilet | 9 (22%) | 29 (21%) | 0.91 | 0.74-1.11 | 0.34 | 33 (22%) | 5 (19%) | 0.72 | 0.28-1.84 | 0.49 |
| Water storage |  |  |  |  |  |  |  |  |  |  |
| Closed with narrow opening | 10 (24%) | 45 (33%) | Ref |  |  | 42 (28%) | 13 (48%) | Ref |  |  |
| Closed with wide opening | 15 (37%) | 36 (27%) | 0.86 | 0.69-1.07 | 0.18 | 47 (31%) | 4 (15%) | **0.33** | **0.12-0.95** | **0.04** |
| Open container | 16 (39%) | 55 (40%) | 0.95 | 0.79-1.13 | 0.55 | 61 (41%) | 10 (37%) | 0.60 | 0.28-1.26 | 0.18 |
| Water treatment |  |  |  |  |  |  |  |  |  |  |
| Any water treatment | 12 (29%) | 38 (28%) | 0.98 | 0.82-1.18 | 0.87 | 42 (28%) | 8 (30%) | 1.07 | 0.50-2.29 | 0.86 |
| Water filter | 9 (22%) | 28 (21%) | 0.98 | 0.80-1.20 | 0.85 | 30 (20%) | 7 (26%) | 1.32 | 0.61-2.90 | 0.48 |
| Boiling | 3 (7%) | 5 (4%) | 0.81 | 0.47-1.39 | 0.44 | 7 (4.7%) | 1 (4%) | 0.81 | 0.12-5.28 | 0.83 |
| Strain through cloth | 1 (%) | 13 (10%) | **1.23** | **1.04-1.46** | **0.02** | 13 (9%) | 1 (4%) | 0.45 | 0.07-3.08 | 0.41 |
| Sunlight | 0 (0%) | 1 (1%) | - |  |  | 1 (1%) | 0 (0%) | - |  |  |
| Chlorine | 0 (0%) | 1 (1%) | - |  |  | 1 (1%) | 0 (0%) | - |  |  |
| Water sources |  |  |  |  |  |  |  |  |  |  |
| Rainwater | 11 (27%) | 45 (33%) | 1.07 | 0.91-1.26 | 0.43 | 47 (31%) | 9 (33%) | 1.08 | 0.52-2.26 | 0.84 |
| Lake or river | 3 (7%) | 9 (7%) | 0.97 | 0.69-1.37 | 0.88 | 9 (6%) | 3 (11%) | 1.72 | 0.60-4.91 | 0.31 |
| Unprotected well | 1 (2%) | 8 (6%) | 1.17 | 0.91-1.49 | 0.22 | 8 (5%) | 1 (4%) | 0.72 | 0.11-4.74 | 0.73 |
| Protected well | 1 (2%) | 6 (4%) | 1.12 | 0.82-1.54 | 0.48 | 7 (5%) | 0 (0%) | - |  |  |
| Bottled water | 18 (44%) | 75 (55%) | 1.11 | 0.94-1.31 | 0.21 | 80 (53%) | 13 (48%) | 0.84 | 0.42-1.68 | 0.62 |
| Shared tap | 1 (2%) | 2 (1%) | 0.87 | 0.39-1.94 | 0.73 | 3 (2%) | 0 (0%) | - |  |  |
| Tube well | 40 (98%) | 133 (98%) | 1.03 | 0.58-1.82 | 0.93 | 146 (97%) | 27 (100%) | - |  |  |
| Piped water | 5 (12%) | 10 (7%) | 0.86 | 0.59-1.24 | 0.41 | 12 (8%) | 3 (11%) | 1.35 | 0.46-3.98 | 0.56 |
| **ANIMAL CONTACT** |  |  |  |  |  |  |  |  |  |  |
| No animals inside house | 3 (7%) | 5 (6%) | 0.94 | 0.65-1.37 | 0.76 | 11 (7%) | 0 (0%) | - |  |  |
| Chickens inside house | 21 (51%) | 83 (61%) | 1.10 | 0.93-1.30 | 0.28 | 87 (58%) | 17 (63%) | 1.19 | 0.58-2.46 | 0.63 |
| Ducks inside house | 3 (7%) | 10 (7%) | 1.00 | 0.73-1.37 | 0.99 | 12 (8%) | 1 (4%) | 0.49 | 0.07-3.31 | 0.46 |
| Goats inside house | 0 (0%) | 7 (5%) | - |  |  | 6 (4%) | 1 (4%) | 0.93 | 0.15-5.96 | 0.94 |
| Cows inside house | 0 (0%) | 0 (0%) | - |  |  | 0 (0%) | 0 (0%) | - |  |  |
| Sheep inside house | 0 (0%) | 0 (0%) | - |  |  | 0 (0%) | 0 (0%) | - |  |  |
| Pigs inside house | 0 (0%) | 0 (0%) | - |  |  | 0 (0%) | 0 (0%) | - |  |  |
| Cats inside house | 37 (90%) | 124 (91%) | 1.03 | 0.76-1.38 | 0.86 | 134 (89%) | 27 (100%) | - |  |  |
| Dogs inside house | 0 (0%) | 1 (1%) | - |  |  | 1 (1%) | 0 (0%) | - |  |  |
| Other animals inside house | 1 (2%) | 3 (2%) | 0.98 | 0.55-1.73 | 0.93 | 4 (3%) | 0 (0%) | - |  |  |
| No animals outside house | 0 (0%) | 0 (0%) | - |  |  | 0 (0%) | 0 (0%) | - |  |  |
| Chickens outside house | 40 (98%) | 125 (92%) | - |  |  | 140 (93%) | 25 (93%) | 0.91 | 0.24-3.40 | 0.89 |
| Ducks outside house | 28 (68%) | 111 (82%) | 1.21 | 0.95-1.55 | 0.12 | 113 (75%) | 26 (96%) | 7.11 | 0.99-50.99 | 0.05 |
| Goats outside house | 29 (71%) | 96 (71%) | 1.00 | 0.84-1.19 | 0.99 | 109 (73%) | 16 (59%) | 0.61 | 0.30-1.22 | 0.16 |
| Cows outside house | 34 (83%) | 122 (90%) | 1.17 | 0.86-1.61 | 0.32 | 130 (87%) | 26 (96%) | 3.50 | 0.50-24.60 | 0.21 |
| Sheep outside house | 1 (2%) | 1 (1%) | 0.65 | 0.16-2.61 | 0.54 | 2 (1%) | 0 (0%) | - |  |  |
| Pigs outside house | 0 (0%) | 2 (1%) | - |  |  | 1 (1%) | 1 (4%) | 3.37 | 0.80-14.13 | 0.10 |
| Cats outside house | 41 (100%) | 135 (99%) | - |  |  | 149 (99%) | 27 (100%) | - |  |  |
| Dogs outside house | 41 (100%) | 134 (99%) | - |  |  | 148 (99%) | 27 (100%) | - |  |  |
| Other animals outside house | 0 (0%) | 1 (1%) | - |  |  | 1 (1%) | 0 (0%) | - |  |  |
| **FOOD CONSUMPTION** |  |  |  |  |  |  |  |  |  |  |
| No animal products | 0 (0%) | 0 (0%) | - |  |  | 0 (0%) | 0 (0%) | - |  |  |
| Eggs | 41 (100%) | 136 (100%) | - |  |  | 150 (100%) | 27 (100%) | - |  |  |
| Dairy | 41 (100%) | 135 (99%) | - |  |  | 149 (99%) | 27 (100%) | - |  |  |
| Chicken | 41 (100%) | 135 (99%) | - |  |  | 149 (99%) | 27 (100%) | - |  |  |
| Beef | 40 (98%) | 124 (91%) | - |  |  | 139 (93%) | 25 (93%) | 0.99 | 0.26-3.74 | 0.99 |
| Goat | 14 (34%) | 54 (40%) | 1.06 | 0.90-1.24 | 0.51 | 55 (37%) | 13 (48%) | 1.49 | 0.74-2.98 | 0.26 |
| Sheep | 0 (0%) | 2 (1%) | - |  |  | 2 (1%) | 0 (0%) | - |  |  |
| Pork | 0 (0%) | 2 (1%) | - |  |  | 2 (1%) | 0 (0%) | - |  |  |
| Fish | 41 (100%) | 131 (96%) | - |  |  | 145 (97%) | 27 (100%) | - |  |  |
| Shellfish | 0 (0%) | 0 (0%) | - |  |  | 0 (0%) | 0 (0%) | - |  |  |
| Raw produce | 41 (100%) | 136 (100%) | - |  |  | 150 (100%) | 27 (100%) | - |  |  |
| **OBSTETRIC HISTORY** |  |  |  |  |  |  |  |  |  |  |
| Prior pregnancy | 28 (68%) | 91 (67%) | 0.99 | 0.83-1.17 | 0.87 | 103 (69%) | 16 (59%) | 0.71 | 0.35-1.43 | 0.34 |
| Prior miscarriage | 23 (56%) | 79 (58%) | 1.02 | 0.83-1.20 | 0.82 | 86 (57%) | 16 (59%) | 1.07 | 0.53-2.17 | 0.85 |
| Prior deliveries | 26 (93%) | 90 (99%) | 2.33 | 0.47-11.65 | 0.30 | 101 (98%) | 15 (94%) | 0.39 | 0.07-2.07 | 0.27 |
| Children at home |  |  |  |  |  |  |  |  |  |  |
| 0-1 | 16 (39%) | 61 (45%) | Ref |  |  | 68 (45%) | 9 (33%) | Ref |  |  |
| 2+ | 25 (61%) | 75 (55%) | 0.95 | 0.81-1.11 | 0.51 | 82 (55%) | 18 (67%) | 1.54 | 0.73-3.24 | 0.26 |
| Prenatal care |  |  |  |  |  |  |  |  |  |  |
| None | 6 (15%) | 29 (21%) | 1.09 | 0.91-1.31 | 0.36 | 28 (19%) | 7 (26%) | 1.56 | 0.69-3.53 | 0.29 |
| 1-3 visits | 28 (68%) | 89 (65%) | Ref |  |  | 102 (68%) | 15 (56%) | Ref |  |  |
| 4 or more visits | 7 (17%) | 18 (13%) | 0.95 | 0.73-1.23 | 0.69 | 20 (13%) | 5 (19%) | 1.56 | 0.62-3.91 | 0.34 |
| Pregnancy complications |  |  |  |  |  |  |  |  |  |  |
| Any complication | 26 (63%) | 78 (57%) | 0.95 | 0.69-1.31 | 0.75 | 85 (57%) | 19 (70%) | 1.35 | 0.46-3.98 | 0.59 |
| Anemia | 22 (54%) | 65 (48%) | 0.95 | 0.90-1.11 | 0.51 | 73 (49%) | 14 (52%) | 1.11 | 0.55-2.24 | 0.76 |
| Urinary infection | 7 (17%) | 25 (18%) | 1.02 | 0.83-1.25 | 0.85 | 25 (17%) | 7 (26%) | 1.59 | 0.73-3.44 | 0.24 |
| Bleeding | 4 (10%) | 13 (10%) | 0.99 | 0.75-1.31 | 0.97 | 14 (9%) | 3 (11%) | 1.18 | 0.39-3.51 | 0.77 |
| Preterm labor | 2 (5%) | 10 (7%) | 1.09 | 0.84-1.43 | 0.52 | 10 (7%) | 2 (7%) | 1.10 | 0.29-4.12 | 0.89 |
| Hypertension | 4 (10%) | 10 (7%) | 0.92 | 0.66-1.30 | 0.65 | 12 (8%) | 2 (7%) | 0.93 | 0.24-3.55 | 0.92 |
| Underweight | 0 (0%) | 3 (2%) | - |  |  | 1 (1%) | 2 (7%) | **4.64** | **1.92-11.2** | **0.01** |
| Hospitalization during pregnancy | 2 (5%) | 19 (14%) | **1.21** | **1.02-1.42** | **0.03** | 14 (9%) | 7 (26%) | **2.60** | **1.25-5.41** | **0.01** |
| Antibiotic use within 30 days | 4 (10%) | 19 (14%) | 1.09 | 0.88-1.34 | 0.43 | 16 (11%) | 7 (26%) | **2.34** | **1.11-4.90** | **0.03** |
| Preterm delivery | 8 (20%) | 46 (34%) | 1.16 | 1.00-1.36 | 0.05 | 44 (29%) | 10 (37%) | 1.34 | 0.66-2.73 | 0.42 |
| Pre-delivery ESBL colonization | N/A |  |  |  |  | 109 (73%) | 27 (100%) | - |  |  |
| Pre-delivery CRO colonization | 0 (0%) | 27 (19.9%) | - |  |  | N/A |  |  |  |  |

Evaluation of community-based exposures with maternal pre-delivery colonization using bivariate regression analyses without adjustment. Results in bold have p<0.05 level. RR = relative risk; ESBL-PB = organisms recovered from agar selective for extended-spectrum beta-lactamase-producing bacteria; CRB = organisms recovered from agar selective for carbapenem resistant bacteria. **-** indicates no data.

#### S5 Table. Bivariate associations of hospital-based exposures with maternal post-delivery ESBL-PB/CRB vaginal and rectal colonization, Faridpur, Bangladesh, 2020

|  | **Post-delivery ESBL-PB** | | | | | **Post-delivery CRB** | | | | |
| --- | --- | --- | --- | --- | --- | --- | --- | --- | --- | --- |
|  | **non-ESBL-PB**  **(N=3)** | **ESBL-PB**  **(N=174)** |  | | | **non-CRB**  **(N=19)** | **CRB**  **(N=158)** |  | | |
|  | **n (%)** | **n (%)** | **RR** | **95% CI** | **p-value** | **n (%)** | **n (%)** | **RR** | **95% CI** | **p-value** |
| **HOSPITAL FACTORS** |  |  |  |  |  |  |  |  |  |  |
| Delivery mode |  |  |  |  |  |  |  |  |  |  |
| Vaginal | 3 (100%) | 34 (20%) | Ref |  |  | 10 (53%) | 27 (17%) | Ref |  |  |
| C-section | 0 (0%) | 140 (81%) | - |  |  | 9 (47%) | 131 (83%) | **1.28** | **1.05-1.57** | **0.02** |
| Duration of hospitalization |  |  |  |  |  |  |  |  |  |  |
| <4 days | 3 (100%) | 86 (49%) | Ref |  |  | 11 (58%) | 78 (49%) | Ref |  |  |
| 4 or more days | 0 (0%) | 88 (51%) | - |  |  | 8 (42%) | 80 (51%) | 1.04 | 0.94-1.15 | 0.48 |
| Mother received antibiotics | 3 (100%) | 171 (98%) | - |  |  | 18 (95%) | 156 (99%) | 1.34 | 0.60-3.01 | 0.47 |
| Mother received antibiotics pre-delivery | 3 (100%) | 155 (89%) | - |  |  | 14 (74%) | 144 (91%) | 1.24 | 0.94-1.63 | 0.13 |
| Birthing management |  |  |  |  |  |  |  |  |  |  |
| None | 0 (0%) | 113 (65%) | - |  |  | 8 (42%) | 105 (67%) | 1.12 | 0.99-1.27 | 0.07 |
| Membrane stripping/sweeping | 3 (100%) | 47 (27%) | - |  |  | 11 (58%) | 39 (25%) | **0.83** | **0.71-0.97** | **0.02** |
| Mechanical cervical ripening | 1 (33%) | 13 (7%) | 0.94 | 0.81-1.09 | 0.41 | 3 (16%) | 11 (7%) | 0.87 | 0.66-1.15 | 0.33 |
| 6 or more vaginal exams | 1 (33%) | 44 (25%) | - |  |  | 8 (42%) | 37 (23%) | 0.90 | 0.78-1.04 | 0.14 |
| Artificial rupture of membranes | 1 (33%) | 18 (10%) | 0.96 | 0.86-1.07 | 0.45 | 5 (26%) | 14 (9%) | 0.91 | 0.61-1.06 | 0.13 |
| Maternal complications |  |  |  |  |  |  |  |  |  |  |
| Any complications | 2 (67%) | 155 (89%) | 1.04 | 0.94-1.15 | 0.46 | 14 (74%) | 143 (91%) | 1.21 | 0.94-1.57 | 0.14 |
| Premature rupture of membranes | 1 (50%) | 40 (26%) | - |  |  | 3 (21%) | 38 (27%) | 1.02 | 0.92-1.14 | 0.66 |
| Obstructed labor | 0 (0%) | 48 (31%) | - |  |  | 2 (14%) | 46 (32%) | 1.08 | 0.99-1.18 | 0.10 |
| Fetal distress | 2 (100%) | 70 (45%) | - |  |  | 2 (14%) | 68 (48%) | **1.13** | **1.03-1.24** | **0.01** |
| Retained placenta | 1 (50%) | 6 (4%) | - |  |  | 1 (7%) | 6 (4%) | 0.94 | 0.69-1.28 | 0.69 |
| Perineal tear | 0 (0%) | 3 (2%) | - |  |  | 0 (0%) | 3 (2%) | - | - |  |
| Maternal infectious complications | 0 (0%) | 4 (3%) | - |  |  | 0 (0%) | 4 (3%) | - | - |  |
| Eclampsia | 0 (0%) | 3 (2%) | - |  |  | 1 (7%) | 2 (1%) | 0.73 | 0.33-1.63 | 0.44 |
| Maternal UTI | 0 (0%) | 4 (100%) | - |  |  | 0 (0%) | 4 (100%) | - | - |  |
| Maternal fever | 0 (0%) | 3 (2%) | - |  |  | 0 (0%) | 3 (2%) | - | - |  |
| Timing of maternal complications |  |  |  |  |  |  |  |  |  |  |
| Before delivery | 0 (0%) | 130 (84%) | - |  |  | 12 (86%) | 118 (83%) | 0.98 | 0.87-1.11 | 0.75 |
| At time of delivery | 2 (100%) | 88 (57%) | - |  |  | 5 (36%) | 85 (59%) | 1.09 | 0.98-1.21 | 0.11 |
| Mother admitted to ICU | 0 (0%) | 9 (5%) | - |  |  | 2 (11%) | 7 (4%) | 0.87 | 0.61-1.23 | 0.42 |
| Mother received third generation cephalosporin antibiotics | 1 (33%) | 115 (66%) | - |  |  | 9 (47%) | 107 (68%) | 1.10 | 0.97-1.25 | 0.12 |
| Pre-delivery ESBL-PB colonization | 1 (33%) | 135 (78%) | - |  |  | 15 (79%) | 121 (77%) | 0.99 | 0.88-1.11 | 0.81 |
| Pre-delivery CRB colonization | 0 (0%) | 27 (16%) | - |  |  | 2 (11%) | 25 (16%) | 1.04 | 0.92-1.18 | 0.48 |

Evaluation of hospital-based exposures with maternal post-delivery colonization using bivariate regression analyses without adjustment. Results in bold have p<0.05 level. RR = relative risk; ESBL-PB = organisms recovered from agar selective for extended-spectrum beta-lactamase-producing bacteria; CRB = organisms recovered from agar selective for carbapenem resistant organisms. **-** indicates no data.

#### S6 Table. Bivariate associations of hospital-based exposures with newborn ESBL-PB/CRB rectal colonization, Faridpur, Bangladesh, 2020

|  | **Newborn ESBL-PB** | | | | | **Newborn CRB** | | | | |
| --- | --- | --- | --- | --- | --- | --- | --- | --- | --- | --- |
|  | **non-ESBL-PB**  **(N=20)** | **ESBL-PB**  **(N=157)** |  | | | **non-CRB**  **(N=49)** | **CRB**  **(N=128)** |  | | |
|  | **n (%)** | **n (%)** | **RR** | **95% CI** | **p-value** | **n (%)** | **n (%)** | **RR** | **95% CI** | **p-value** |
| **HOSPITAL FACTORS** |  |  |  |  |  |  |  |  |  |  |
| Delivery mode |  |  |  |  |  |  |  |  |  |  |
| Vaginal | 11 (55%) | 26 (17%) | Ref |  |  | 20 (41%) | 17 (13%) | Ref |  |  |
| C-section | 9 (45%) | 131 (83%) | **1.33** | **1.07-1.65** | **0.01** | 29 (59%) | 111 (87%) | **1.73** | **1.20-2.47** | **0.00** |
| Low birthweight | 4 (20%) | 25 (16%) | 0.97 | 0.83-1.13 | 0.67 | 9 (18%) | 20 (16%) | 0.95 | 0.73-1.23 | 0.68 |
| Duration of hospitalization |  |  |  |  |  |  |  |  |  |  |
| <4 days | 16 (80%) | 97 (62%) | Ref |  |  | 34 (69%) | 79 (62%) | Ref |  |  |
| 4 or more days | 4 (20%) | 60 (38%) | 1.09 | 0.99-1.20 | 0.08 | 15 (31%) | 49 (38%) | 1.10 | 0.91-1.31 | 0.33 |
| Postnatal management (within 1 hr) |  |  |  |  |  |  |  |  |  |  |
| Cleared airways | 20 (100%) | 156 (99%) | - |  |  | 49 (100%) | 127 (99%) | - |  |  |
| Measured weight and temperature | 17 (85%) | 156 (99%) | 3.61 | 0.66-19.8 | 0.14 | 46 (94%) | 127 (99%) | 2.94 | 0.53-16.1 | 0.22 |
| Wiped | 20 (100%) | 156 (99%) | - |  |  | 49 (100%) | 127 (99%) | - |  |  |
| Skin-to-skin or kangaroo care | 16 (80%) | 110 (70%) | 0.95 | 0.85-1.05 | 0.31 | 35 (71%) | 91 (71%) | 1.00 | 0.81-1.22 | 0.97 |
| Initial feed | 18 (90%) | 153 (98%) | 1.34 | 0.76-2.37 | 0.31 | 47 (96%) | 124 (97%) | 1.09 | 0.61-1.93 | 0.77 |
| Administered vitamin K | 12 (60%) | 86 (55%) | 0.98 | 0.88-1.08 | 0.66 | 25 (51%) | 73 (57%) | 1.07 | 0.89-1.29 | 0.48 |
| Resuscitation | 6 (30%) | 34 (22%) | 0.95 | 0.82-1.09 | 0.45 | 10 (20%) | 30 (23%) | 1.05 | 0.85-1.29 | 0.66 |
| Artificial ventilation | 1 (5%) | 1 (1%) | 0.56 | 0.14-2.25 | 0.42 | 1 (2%) | 1 (1%) | 0.69 | 0.17-2.77 | 0.60 |
| Delayed clamping of umbilical cord | 0 (0%) | 1 (1%) | - |  |  | 0 (0%) | 1 (1%) | - |  |  |
| Sterile cutting of umbilical cord | 20 (100%) | 156 (99%) | - |  |  | 49 (100%) | 127 (99%) | - |  |  |
| Treatment of umbilical cord with chlorhexidine | 20 (100%) | 156 (99%) | - |  |  | 49 (100%) | 127 (99%) | - |  |  |
| Complications |  |  |  |  |  |  |  |  |  |  |
| Any complications | 1 (5%) | 11 (7%) | 1.04 | 0.87-1.24 | 0.70 | 3 (6%) | 9 (7%) | 1.04 | 0.74-1.46 | 0.82 |
| Asphyxia | 0 (0%) | 2 (18%) | - |  |  | 0 (0%) | 2 (22%) | - |  |  |
| Poor feeding | 1 (100%) | 5 (46%) | - |  |  | 1 (33%) | 5 (56%) | 1.25 | 0.52-2.52 | 0.53 |
| Sepsis | 0 (0%) | 3 (100%) | - |  |  | 0 (0%) | 3 (100%) | - |  |  |
| Admission to SCANU | 2 (10%) | 7 (4%) | 0.87 | 0.61-1.24 | 0.45 | 3 (6%) | 6 (5%) | 0.92 | 0.57-1.47 | 0.72 |
| Baby received antibiotics | 1 (5%) | 8 (5%) | 1.00 | 0.79-1.27 | 0.99 | 3 (6%) | 6 (5%) | 0.92 | 0.57-1.47 | 0.72 |
| Mother received antibiotics pre-delivery | 15 (75%) | 143 (91%) | 1.22 | 0.93-1.62 | 0.14 | 41 (84%) | 117 (91%) | 1.28 | 0.86-1.90 | 0.22 |
| Mother received third generation cephalosporin antibiotics | 11 (55%) | 105 (67%) | 1.06 | 0.94-1.20 | 0.33 | 30 (61%) | 86 (67%) | 1.08 | 0.88-1.32 | 0.47 |
| Maternal pre-delivery ESBL-PB colonization | 16 (80%) | 120 (76%) | 0.98 | 0.87-1.10 | 0.71 | 37 (76%) | 99 (77%) | 1.03 | 0.82-1.29 | 0.80 |
| Maternal pre-delivery CRB colonization | 1 (5%) | 26 (17%) | 1.10 | 1.00-1.21 | 0.05 | 6 (12%) | 21 (16%) | 1.09 | 0.87-1.37 | 0.45 |
| Maternal post-delivery ESBL-PB colonization | 18 (90%) | 156 (99%) | 2.69 | 0.54-13.4 | 0.23 | 46 (94%) | 128 (100%) | - |  |  |
| Maternal post-delivery CRB colonization | 14 (70%) | 144 (92%) | 1.33 | 0.98-1.82 | 0.07 | 36 (74%) | 122 (95%) | **2.45** | **1.25-4.77** | **0.01** |

Evaluation of hospital-based exposures with newborn colonization using bivariate regression analyses without adjustment. Results in bold have p<0.05 level. RR = relative risk; ESBL-PB = organisms recovered from agar selective for extended-spectrum beta-lactamase-producing bacteria; CRB = organisms recovered from agar selective for carbapenem resistant bacteria. - indicates no data.

### Supplemental Figures

#### S1 Fig. Causal pathway

**
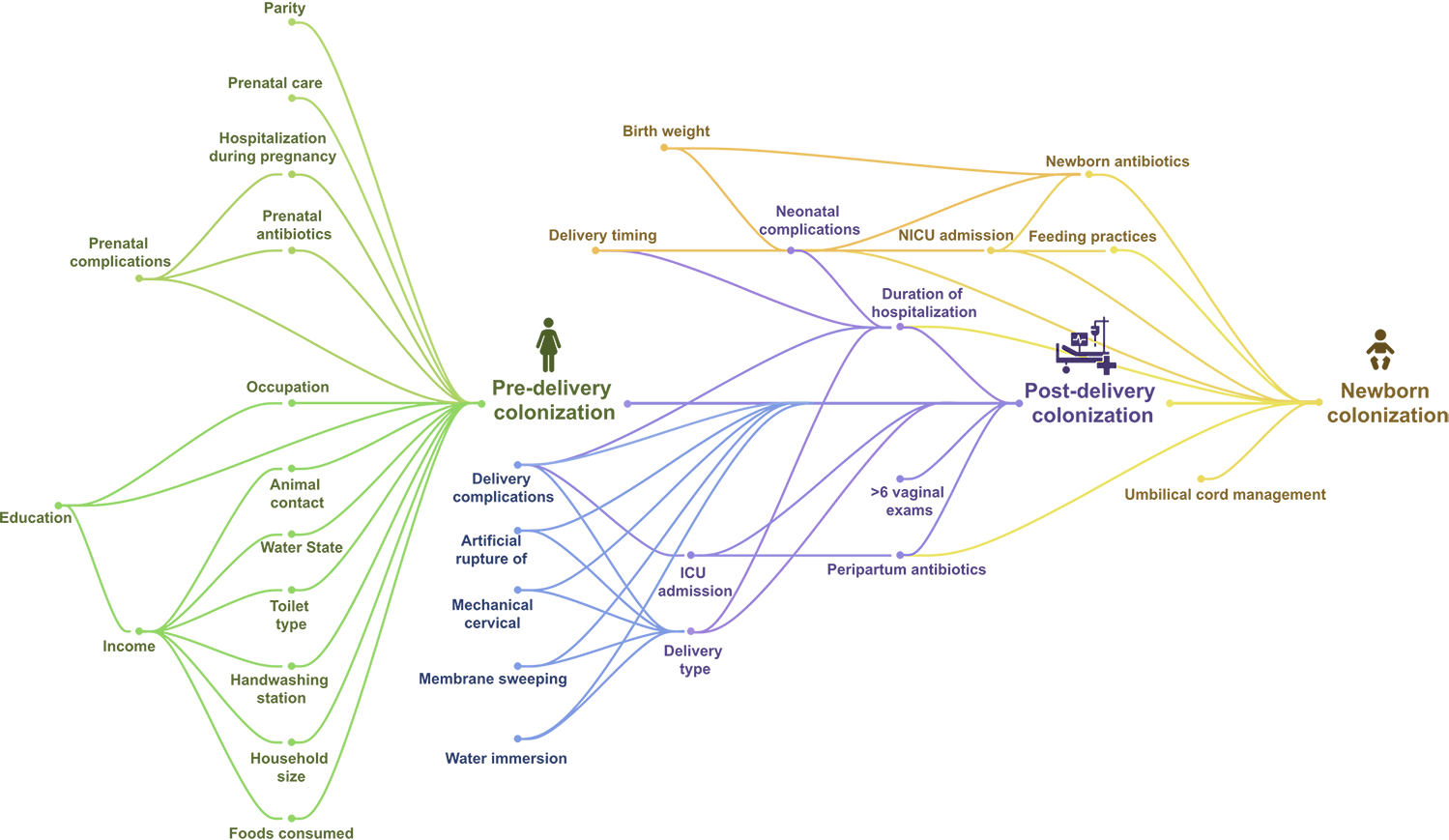
**

A proposed causal pathway examining the relationship between community- and hospital-based factors and colonization with antimicrobial resistant organisms.

#### S2 Fig. Bacterial growth from different environmental surfaces

**
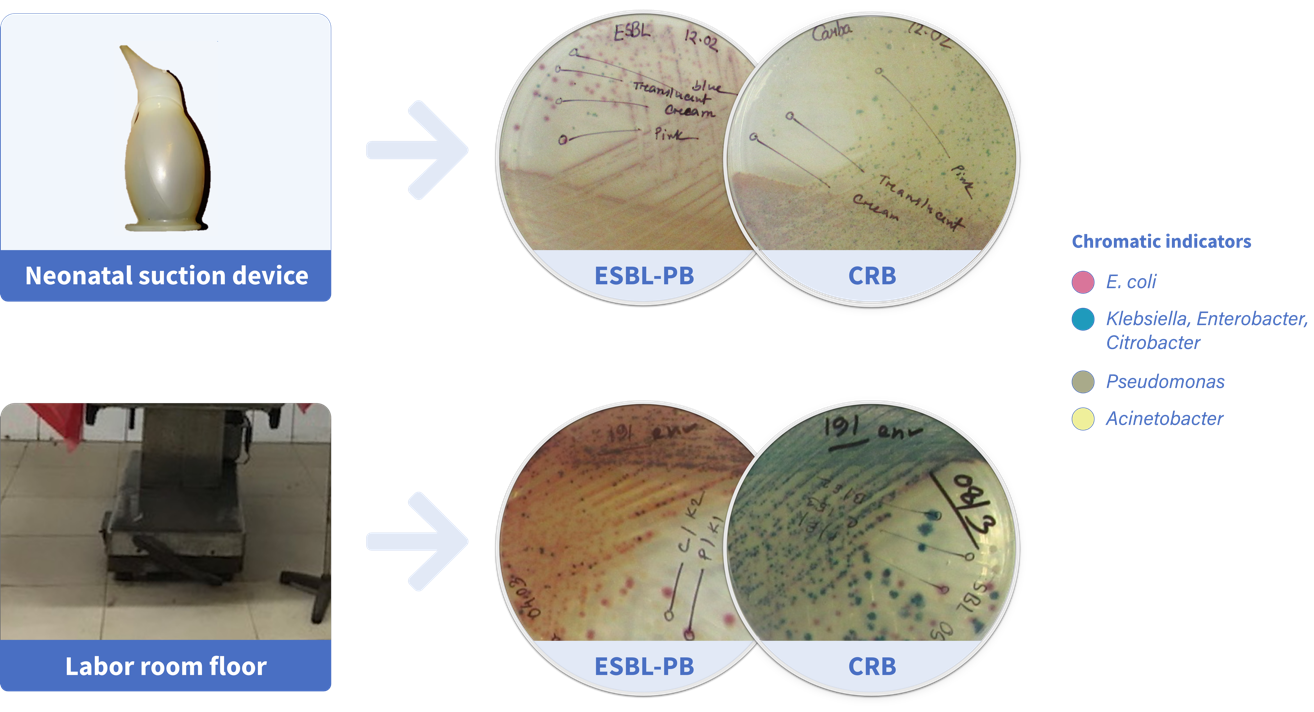
**

The above panels show the bacteriologic results from some of the environmental samples. The top panel demonstrates 4 separate ESBL-PB colony types and 3 CRB colony types. The bottom panel reveals a similarly wide diversity of AMR organisms isolated from the labor room floor. ESBL-PB = organisms recovered from agar selective for extended-spectrum beta-lactamase-producing bacteria; CRB = organisms recovered from agar selective for carbapenem resistant bacteria.

#### S3 Fig. Environmental contamination with ESBL-PB/CRB before and during COVID-19


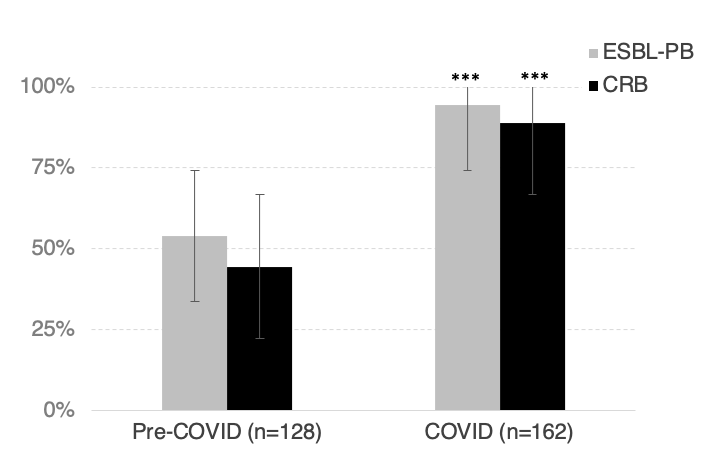


Comparison of environmental contamination with antimicrobial resistant organisms before and during the COVID-19 pandemic using a test of proportions. ESBL = organisms recovered from agar selective for extended-spectrum beta-lactamase-producing organisms; CRB = organisms recovered from agar selective for carbapenem resistant organisms. ****significant at the p<0.0001 level*
